# Amyotrophic Lateral Sclerosis: A Focus on Disease Progression

**DOI:** 10.1155/2014/925101

**Published:** 2014-08-03

**Authors:** Ana C. Calvo, Raquel Manzano, Deise M. F. Mendonça, María J. Muñoz, Pilar Zaragoza, Rosario Osta

**Affiliations:** ^1^LAGENBIO-I3A, Veterinary Faculty of Zaragoza, Aragonese Institute of Health Sciences (IACS), University of Zaragoza, Miguel Servet 177, 50013 Zaragoza, Spain; ^2^Laboratory of Neurobiology of Degenerative Diseases of the Nervous System, Biosciences Department, Federal University of Sergipe, Avenida Vereador Olimpio Grande, s/n, Centro, 49500-000 Itabaiana, SE, Brazil

## Abstract

Since amyotrophic lateral sclerosis (ALS) was discovered and described in 1869 as a neurodegenerative disease in which motor neuron death is induced, a wide range of biomarkers have been selected to identify therapeutic targets. ALS shares altered molecular pathways with other neurodegenerative diseases, such as Alzheimer's, Huntington's, and Parkinson's diseases. However, the molecular targets that directly influence its aggressive nature remain unknown. What is the first link in the neurodegenerative chain of ALS that makes this disease so peculiar? In this review, we will discuss the progression of the disease from the viewpoint of the potential biomarkers described to date in human and animal model samples. Finally, we will consider potential therapeutic strategies for ALS treatment and future, innovative perspectives.

## 1. Pathophysiology, Epidemiology, and Essential Features of Amyotrophic Lateral Sclerosis

Amyotrophic Lateral Sclerosis (ALS) is an adult-onset, devastating, neurodegenerative disease characterized by the loss of cortical, brain stem, and spinal motor neurons. The average survival from symptom onset is approximately 3 to 5 years, although some patients survive longer and exhibit a slower disease progression. In accordance with the revised El Escorial criteria [1], both the upper motor neurons and the lower motor neurons degenerate or die in ALS, and, as a consequence, the communication between the neuron and muscle is lost, prompting progressive muscle weakening and the appearance of fasciculations. In the later disease stages, the patients become paralyzed and up to 50% of ALS patients can show cognitive impairment, particularly implicating more severe executive dysfunction and mild memory decline [2–4].

ALS, which is one of the most common motor neuron degenerative diseases, typically strikes adults during midlife. Although ALS cases have been detected all over the world, interestingly, the prevalence of the geographic loci of the Western Pacific form of ALS is 50–100 times higher than elsewhere in the world. The population of the Chamorro people of Guam and Marianas is very well known. One of the first studies performed in this population in 1957 described a genetic origin of ALS associated with Parkinsonism and dementia independent of environmental changes [5]. However, recent studies have noted a decreased incidence in these areas over the past 40 years [6].

The majority of ALS cases are sporadic (SALS). Approximately 5–10% of cases are the familial form of the disease (FALS), in which 20% have a SOD1 gene mutation and approximately 2–5% have mutations in the TARDBP gene (TAR DNA binding-protein, TDP-43) [7]. Additionally, mutations in this gene also occur in SALS [6]. Other genes, such as fusion in malignant liposarcoma/translocated in liposarcoma (FUS/TLS), angiogenin (ANG), the vesicle-associated membrane protein-associated protein B (VAPB), senataxin (SETX), dynactin, and the most recently discovered gene, a hexanucleotide repeat expansion in* C9ORF72*, have been identified in ALS patients [8, 9]. These corresponding gene mutations are responsible for ALS1 to ALS8, XALS, ALS/FTD1 and ALS/FTD2 disease forms [10, 11]. In particular,* C9ORF72* gene has been defined as the most common mutation in SALS and FALS, representing up to 6% and up to 40% respectively, with or without frontotemporal dementia (FTD). This expansion shows a variable percentage of penetrance, which is directly correlated to the age of the patient. From the molecular point of view, the mRNA transcript of the* C9ORF72* expansion diminishes the pool of RNA binding proteins, deregulating finally the RNA metabolism [12]. Other candidate genes that have been described in ALS genome association studies, such as neurofilament, peripherin [13], vascular endothelial growth factor (VEGF), angiogenin, survival motor neuron (SMN), and hemochromatosis (HFE) [14], are also summarized in Table 1.

Most FALS cases have an autosomal dominant inheritance pattern, although autosomal recessive patterns have also been reported. For instance, the D90A mutation in the SOD1 gene was detected in 3 of 28 German families [15]; mutations in the ALS2 gene are linked to a locus on chromosome 2q33, which has been described as a rare juvenile form of ALS, and mutations in the ALS5 gene, linked to chromosome 15q15.1-q21.1, are the most prevalent form of autosomal recessive ALS, especially in families from Asia, North Africa, and Germany [16]. Furthermore, the coexistence of dominantly inherited FALS with other syndromes, such as FTD and Parkinson's disease, is also described. The first case in which ALS coexisted with FTD was first reported in 1975 [17] and was linked to a locus on chromosome 9q21-q22, although the fact that different loci have also been linked to this ALS-FTD phenotype suggests its genetic heterogeneity [16]. Another ALS phenotype, FTD and Parkinson's disease (FTDP), was discovered in North America and was linked to chromosome 17, showing clinical heterogeneity as in all cases in which mutations in the microtubule-associated protein tau gene (MAPT) located on chromosome 17q21 were described. Moreover, genetic heterogeneity was also found when MAPT mutations were not common among all patients who had the FTDP phenotype [16, 18].

The onset age of FALS, which follows a normal Gaussian distribution, is a decade earlier than the onset age of SALS, which has an age-dependent incidence. The incidence of SALS is 1.89 people per 100,000/year and the prevalence is 5.2 people per 100,000. The median age of onset is approximately 60 years. The median survival ranges between 3 and 5 years from symptom onset, which varies between 55 and 65 years with a median age of 64 years [6, 16]. Onset before age 30, called juvenile sporadic onset, is only found in 5% of cases. The life-time risk of SALS by the age of 70 has been accurately estimated to be 1 in 400 [6, 19].

Another important consideration is that many studies have demonstrated that a slight excess of males are affected with SALS compared to females, with a M : F ratio of approximately 1.5 : 1, which may be due to protective hormones in women. However, recent data suggest equality in this gender ratio. Furthermore, bulbar onset is more common in women and in older age groups and it is characterized by the presence of dysarthria and dysphagia for solid and liquids and limb symptoms that can develop almost simultaneously with the bulbar symptoms, within 1 or 2 years in most cases. Paralysis is a progressive process that leads to death within 2-3 years for bulbar onset cases and within 3–5 years for limb onset cases [6].

A precise patient diagnosis is difficult, even for an experienced clinician, because there are a wide range of motor neuron diseases, such as FALS/SALS, spinal muscular atrophy (SMA), hereditary spastic paraplegia (HSP), primary lateral sclerosis (PLS), spinobulbar muscular atrophy (SBMA), or Kennedy disease, that share common and heterogeneous symptoms, such as weakness, spastic paralysis, or both, reflecting a functional loss of upper and/or lower motor neurons [20, 21]. ALS also includes dysfunction and loss of both upper and lower motor neurons and spasticity may gradually become present in the weakened limb, which can affect manual dexterity and gait [6]. By definition, there should be no autonomic, sensory, or cognitive involvement [22]. Furthermore, the death of motor neurons tends to occur in the distal segments toward the axial body region and the clinical manifestation corresponds to the motor neuron degeneration pattern. However, the evolution of the degeneration is unique among ALS patients and the clinical symptoms reflect these variations. Histopathological studies have revealed cytoplasmic aggregate inclusions in the somata and axons of surviving motor neurons, extensive gliosis, and the presence of atrophic neurons or extensive neuronal loss [21]. Moreover, ubiquitin, neurofilament subunits, and TDP-43 are common proteins found within the inclusion bodies. Particularly, TDP-43 was found in the neuronal inclusions of both ALS and ALS-FTD patients and it may be used to differentiate between SOD1 and non-SOD1 ALS cases [21].

## 2. Guidelines for Disease Diagnosis 

**Figure 1:**
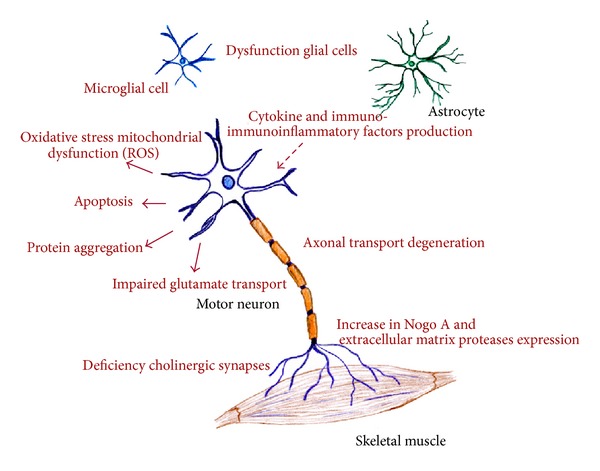
Schematic representation of the different molecular pathways altered in amyotrophic lateral sclerosis. Although the trigger for neurodegeneration remains unknown, all of these deregulated mechanisms prompt motor neuron death.

ALS is a syndrome that appears to result from a complex array of factors, including oxidative stress, mitochondrial dysfunction, endoplasmic reticulum stress, dysregulated transcription and RNA processing, dysregulated endosomal trafficking, impaired axonal transport, protein aggregation, excitotoxicity, apoptosis, inflammation, and genetic susceptibility. All of these factors, or just some of them, can contribute in different ways to the pathogenesis of the disease (Figure 1). The potential role for glutamate excitotoxicity in the pathophysiological process of FALS and SALS has been thoroughly described during the past decade. An excessive activation of the postsynaptic receptors N-methyl-d-aspartate (NMDA) and *α*-amino-3-hydroxy-5-methyl-4-isoxazolepropionic (AMPA) and a significant reduction in the expression of glutamate transporters (EAAT2, GLT-1), which remove glutamate from the synaptic cleft, result in an increased intracellular Ca^2+^ concentration, which prompts glutamate excitotoxicity and, in later stages, motor neuron death due to the production of free radicals [16, 23, 24]. From an anatomical perspective, Eisen and colleagues proposed that the dysfunction of upper motor neuron or corticomotor neurons could induce deregulation in glutamate metabolism and, as a result, an anterior horn cell degeneration in an anterograde manner, which is known as the “*dying forward hypothesis.*” This hypothesis has been supported by studies demonstrating a cortical hyperexcitability in ALS patients, which seemed to be an early feature in both FALS and SALS [25, 26].

Despite the numerous studies that have attempted to identify specific gene/protein targets exclusive to ALS and characteristic of both FALS and SALS, the prognosis of the disease remains poor. For this reason, the guidelines for ALS diagnosis should be accurately defined. These guidelines were first proposed by Lambert and colleagues [27], who considered that neurophysiologic symptoms, especially fasciculations during electromyography (EMG), played an essential role in ALS diagnosis and in the exclusion of other peripheral neuromuscular pathologies. In particular, Lambert stated that the presence of fasciculations on EMG recordings was an essential feature for ALS diagnosis [27]. In 1990, the limits of ALS were defined for the first time in the “El Escorial” workshop for the purpose of facilitating clinical studies and trials all over the world. The World Federation of Neurology Research Group on Motor Neuron Diseases developed the “El Escorial” diagnostic criteria in 1994. Four years later, in the revised “Airlie House” criteria, the diagnosis of patients was divided into four categories: “clinically definite,” “clinically probable,” “clinically probable-laboratory supported,” and “clinically possible” [6, 28]. Indeed, these criteria appeared to be more useful for research purposes and therapeutic trials rather than clinical trials because some patients with clinically obvious ALS failed to be categorized other than clinically possible [28]. In fact, fasciculation potentials were not accepted as evidence of active denervation, and their absence raised diagnostic doubts [28]. A new criteria called “Awaji-Shima” was proposed at an international symposium in December 2006 during a consensus conference in Awaji-shima, Japan, sponsored by the International Federation of Clinical Neurophysiology [27]. The “Awaji-Shima” criteria allowed EMG and clinical abnormalities to be combined in the assignment of the diagnostic category of the patient based on three categories: “clinically possible ALS,” “clinically probable ALS,” and “clinically definite ALS”. In both criteria, evidence of active and chronic denervation is required to be confirmed by the presence of positive sharp waves and fibrillation potentials and by the presence of long duration, large amplitude, polyphasic, and unstable motor unit potentials and decreased motor unit recruitment. In the “Awaji-Shima” criteria, the presence of fasciculation potentials denotes acute denervation, which is equivalent to fibrillation potentials and positive waves according to the Lambert criteria. These abnormalities must be present in at least two muscles corresponding to the cervical and lumbosacral spinal cord affected regions and in one muscle corresponding to the brainstem and thoracic cord affected regions [27, 29]. Therefore, the accepted fasciculations should be complex fasciculation potentials that occur in the presence of unstable motor unit potentials in suspected ALS cases [27]. Carvalho and Swash found a 95% increase in the sensitivity of the diagnostic criteria for definite ALS, especially for bulbar onset patients and for patients with “El Escorial” clinically possible ALS, without loss of specificity compared to the “El Escorial” criteria [29]. The use of the “Awaji-Shima” criteria reduces periods of diagnostic uncertainty and repetitive testing and enables earlier recruitment into clinical trials [30]. Another useful instrument for the evaluation of the functional status of ALS patients is the revised ALS Functional Rating Scale (ALSFRS-r). This parameter can be used to monitor the functional state of the patient over time and it includes the measurement of speech, salivation, swallowing, handwriting, cutting food and handling utensils, dressing and hygiene, turning in bed and adjusting bed clothes, walking, climbing stairs, and breathing. The progression of ALSFRS-r is strongly related to survival and ALS prognosis [31].

## 3. Potential ALS Biomarkers Corresponding to Disease Progression

According to the Biomarkers Definitions Working Group, a biomarker, which has to be objectively measured and evaluated, is “an indicator of normal biological processes, pathogenic processes, or pharmacologic responses to a therapeutic intervention” (DWG 2001) and “an indicator of functional and structural changes in organs and cells.” Therefore, biomarkers can also be considered potential therapeutic molecular targets [32].

On the basis of their specific application to disease detection, biomarkers can be divided into three main categories [32]: screening biomarkers, which may predict the potential occurrence of a disease in asymptomatic patients; diagnostic biomarkers, which are used to make predictions about patients suspected of having the disease; and prognostic biomarkers that are applied to predict the outcome of a patient suffering from the disease. In the particular case of ALS, during the last three decades, a wide range of potential target molecules involved in different molecular pathways have been described as possible biomarkers and these potential protein biomarkers could most likely fit in at least one of the aforementioned categories. Which molecular biomarkers are the key elements that induce neurodegeneration in ALS in each stage of the disease?

Particularly useful for prognosis is the accurate identification of the stage of the disease. Although the main causes of the ALS disease remain unknown, the stage of the disease can be estimated using the ALSFRS-r, as previously mentioned. The four main domains in the ALSFRS-r, swallowing, walking/self-care, communicating, and breathing, have been well characterized. Stages were defined as stage 0 (functional involvement but no loss of independence on any domain), stages 1–4 (number of domains in which independence was lost), and stage 5 (death). However, recent studies devised an algorithm to convert the ALSFRS-r score into clinical stage, with an intraclass correlation coefficient of 0.92. This algorithm provided a useful and reliable clinical stage using the ALSFRS-r recorded [33]. Other ALS staging systems has been also proposed. This is the case of the ALS Milano-Torino Staging which combines the ALSFRS, quality of care (QOC), and quality of life (QOL) measures. This system correlated well with assessments of function, QOL, and health service costs [34]. More recently, another potential tool for predicting disease progression is the multimodal magnetic resonance imaging (MRI) of the spinal cord. This neuroimaging tool has been mostly used in the study of neurodegenerative changes in the brain, and therefore the focus of these studies on ALS was mainly the upper motor neuron involvement. Notwithstanding, spinal cord MRI, apart from its technical limitations, gives the opportunity to extend the study to the lower motor neuron area. In particular, spinal cord cross-sectional area and the magnetization transfer ratio (MTR) were found reliable and sensitive markers of the disease progression in 29 patients with probable or definite ALS. The atrophy rate of change obtained by these markers strongly correlated with the rates of change in the arm ALSFRS-r and manual muscle testing (MMT) subscores [35].

**Figure 2:**
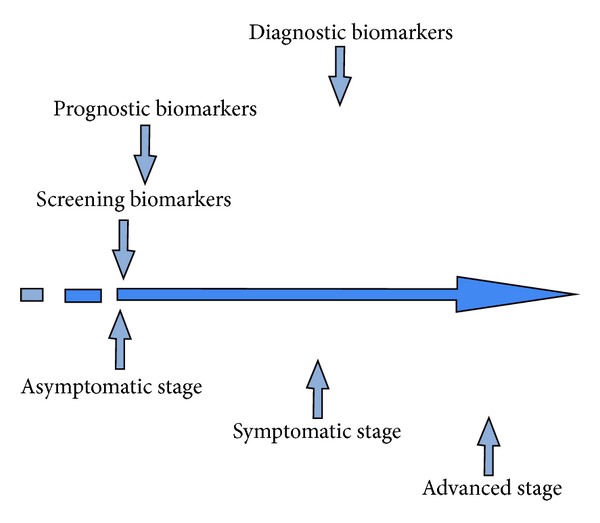
Time course of disease progression and identification of related potential biomarkers involved in each stage. Reliable biomarkers can be helpful in the prediction of different disease stages and will facilitate the application of accurate therapeutic treatments.

In the literature and from the molecular point of view a range of potential biomarkers of disease progression have been defined. In the asymptomatic stage, no visible signs can be detected. In the symptomatic stage, the first clinical symptoms and functional deficits start to appear. The advanced or terminal stage corresponds to the end-stage of the disease. Although a wide range of molecular targets and potential biomarkers have been studied and their relationship to a specific disease stage has also been shown, few molecular targets have been defined as potential ALS biomarkers based on their relevant role in the prognosis and diagnosis of the disease (Figure 2).

The asymptomatic stage or early asymptomatic stage is the most critical stage and an accurate identification of potential biomarkers in the asymptomatic stage could enable early disease prognosis. More than 18 murine ALS models have contributed not only to a better understanding of the disease but also to the search of potential therapeutic strategies. Nogo, also known as Neurite outgrowth inhibitor or Reticulon-4, was one of the first potential biomarkers suggested in early asymptomatic transgenic SOD1^G86R^ mice [36, 37]. Nogo is a member of the myelin-associated proteins, which possess axonal growth inhibitory activity. Three isoforms of this Neurite outgrowth inhibitor have been identified, Nogo A, Nogo B, and Nogo C. In particular, blocking Nogo A during neuronal damage has been proposed as a potential therapeutic strategy for autoimmune diseases, such as multiple sclerosis [38]. Interestingly, Nogo could also have a central role in ALS. Nogo is upregulated in the lumbar spinal cord of asymptomatic transgenic SOD1^G86R^ mice and has three isoforms whose expression levels peak at different stages before the onset of pathological symptoms [36]. Furthermore, in the skeletal muscle from these transgenic mice, Nogo A expression levels increased close to the onset of the disease and Nogo C expression levels decreased before the first pathological signs in parallel with the overexpression of AChR*α*, which is a marker of denervation [36]. These findings suggested for the first time that the differential patterns of Nogo A and Nogo C could be relevant to the asymptomatic stage of the disease and the same profile pattern was observed in postmortem muscle samples and in muscle biopsies from ALS patients [36]. Another relevant result of these findings was that Nogo A and Nogo B protein levels were systematically increased exclusively in ALS patients and they correlated with the severity of motor impairment assessed by the ALS functional rating scale (ALSFRS) [39], which opened the door to the study of this potential biomarker in unexplored tissues. In accordance with muscle weakness, reduced levels of Vgf nerve growth factor inducible peptide, a member of the chromogranin/secretogranin family of proteins, in the cerebrospinal fluid (CSF) were proposed as a useful indicator of disease progression [40]. The hypothesis suggested by Zhao and coworkers was based on the reduced levels of Vgf found in CSF, serum, and spinal cord motor neurons from ALS patients, resembling the same findings in the asymptomatic transgenic SOD1^G93A^ mice. Consequently, the downregulation of Vgf levels in spinal cord motor neurons could promote neurodegeneration, prompting NMDA and AMPA excitotoxicity injury [40]. Similarly, upregulated levels of glial fibrillary acidic protein (GFAP) were also found in the spinal cords of 25–30-day-old transgenic SOD1^G93A^ mice and this upregulation was associated with the onset of paralysis at the symptomatic stage [41].

Regarding the symptomatic stage, a wide range of ALS biomarkers have been proposed in different tissues. Numerous studies have demonstrated the potential nature of relevant biomarkers in the spinal cord, which is one of the most affected tissues. Monitoring the levels of 5-methyltetrahydrofolate (5-MTHF), folic acid, and homocysteine (Hcy) could provide useful information about the early signs of the disease. Decreased levels of 5-MTHF were found in the plasma, spinal cord, and cortex during the early presymptomatic transgenic mice SOD1^G93A^ prior to folic acid reduction. Interestingly, the deficiency observed in 5-MTHF and folic acid was followed by an increase in Hcy levels after motor symptoms appeared in this animal model, suggesting that 5-MTHF could be a potential biomarker in the early disease stages [42]. With a clear connection to the spinal cord and brain, microglial cells are also extremely sensitive to pathological changes in the CNS. In fact, a dysregulation of these cells can give rise to neurological diseases. Interestingly, enhanced expression of the growth factor progranulin (PGRN) in microglia from transgenic SOD1 mice during disease progression was observed in the spinal cord and CSF. Consequently, the upregulation of PGRN, which begins during the symptomatic stage, could serve as a marker of microglial response corresponding to disease progression [43]. In agreement with this study, there is growing evidence for the role of glial cells in the initiation of the disease. A deficit in the number of microglial cells was reported in the spinal cord from transgenic SOD1^G93A^ mice at 30 days of age, reflecting an intrinsic alteration of this cell population that is tightly connected to an immune deficit even before the early symptomatic stage [44]. However, activated microglial cells could produce nitric oxide (NO) during symptomatic and late stages, contributing to the neurodegenerative processes of the disease. A significant upregulation of isoenzyme inducible NO synthase (iNOS) was observed in spinal cord glial cells from transgenic SOD1^G93A^ mice during the early symptomatic and end-stages, prompting the formation of NO and other reactive species, favoring an oxidative stress state in motor neurons. Contrary to iNOS regulation, the neuronal NO synthase isoform (nNOS), another source of NO, showed a downregulation in the spinal cord of SOD1^G93A^ mice over the course of ALS. This downregulation in nNOS enzymatic activity was observed in parallel to the reduced number of motor neurons, suggesting that nNOS activity could also be a useful marker of spinal cord neurodegeneration in this mouse model [45].

Recent studies have shown that *T*_2_-weighted magnetic resonance imaging (MRI) was sensitive to pathologic changes in brainstem nuclei from transgenic SOD1^G93A^ mice at symptom onset. Therefore, *T*_2_-weighted MRI has been proposed as a leading candidate biomarker of disease progression in this animal model. Furthermore, the results obtained by this noninvasive assessment correlated with histologic measures of vacuolation and GFAP and Iba1 staining, which were significantly greater during the symptomatic to late stage of the disease [46].

Particularly interesting is the alteration of the blood brain barrier (BBB) and blood spinal cord barrier (BSCB) during disease progression. The BBB/BSCB impairment could serve as a hallmark preceding clinical symptoms. In SOD1^G93A^ rats, decreased mRNA levels of the tight junction proteins zonula occludens (ZO-1), occludin, and agrin were observed during the symptomatic stage, pointing to an alteration in the BBB/BSCB permeability and development. The BBB/BSCB integrity was also found to be altered, possibly due to the mutant SOD1 toxicity on endothelial cells and other factors released as a direct consequence of the induced inflammation during the last stage of the disease [47]. However, for SOD1^G93A^ mice, neither BBB leakage nor upregulation of VCAM-1 expression has been proposed as a substantial aspect of pathology [46]. The presence of different molecules in the blood has a clear connection with BBB and BSCB integrity and these molecules come from other damaged tissues during disease progression. One example is matrix metalloproteinase-9 (MMP-9) levels in the serum. Serum MMP-9 activity was significantly elevated in presymptomatic transgenic SOD1^G93A^ mice, preceding a later decrease of its activity in later disease stages. The upregulation of MMP-9 during early disease stages could indicate a response to spinal cord pathology and other damaged tissues, such as skeletal muscles and peripheral nerves. Therefore, circulating MMP-9 activity in the blood could reflect disease progression in this animal model [48]. However, previous studies on the CSF of ALS patients did not reveal a significant upregulation of MMP-9 levels [49].

Other useful diagnostic ALS biomarkers have been described in more accessible tissues, such as blood or urine samples. Closely related to the inflammatory response, 11,15-dioxo-9-hydroxy-,2,3,4,5-tetranorprostan-1,20-dioic acid (tPGDM), a metabolite of prostaglandin D2, could also play an important role in the presymptomatic stages and evaluation of ALS. Prostaglandins could mediate neurodegeneration in ALS and muscle dystrophy. A significant correlation between urinary tPGDM concentration and ALS progression could provide a presymptomatic diagnosis of the disease. Furthermore, the accuracy of this analysis was improved when the combination of urinary tPGDM and creatinine levels was monitored during disease progression [50]. Another attractive candidate that can be measured in an accessible tissue, such as blood, is the heavily phosphorylated axonal form of neurofilament H (pNF-H). pNF-H levels were upregulated in the blood from rodent ALS models and ALS patients. Interestingly, this upregulation correlated with a decline in ALSFRS-R score in ALS patients, supporting the possibility of using this molecule as a biomarker for disease prognosis [51].

Survival has been used as a relevant factor, mainly in transgenic animal models where disease progression is rapid. In skeletal muscle biopsies from transgenic SOD1^G93A^ mice, five genes (myocyte enhancer factor 2C (*Mef2c*), glutathione reductase (*Gsr*), collagen, type XIX, alpha 1 (*Col19a1*), calmodulin 1 (*Calm1*), and sorting nexin 10 (*Snx10*)) have been proposed as potential genetic biomarkers of longevity because their expression levels in skeletal muscle during disease progression could be used to predict the longevity of the animals. In light of this, In light of this, animals that displayed downregulated leves of these genes during the early symptomatic stage, showed a longer survival rate than animals that presented an upregulation in these levels. Therefore, these animals possibly exhibited a higher regenerative capacity of skeletal muscle [52].

In the advanced disease stage, identifying which target molecule is involved in the causative pathways of the disease and which is a bystander phenomenon is very difficult. At this step, the identification of ALS-specific protein biomarkers in the CSF or serum could provide invaluable information about the nature of the degenerative ALS process because CSF or blood can be a source of proteins and protein fragments released from affected neuronal and nonneuronal cells. An example of protein biomarkers with diagnostic predictive value comes from a study based on mass spectrometry (surface enhanced laser desorption/ionization time-of-flight mass spectrometry, SELDI-TOF-MS) of transthyretin, cystatin C, and carboxy-terminal fragment of neuroendocrine protein 7B2 (7B2CT) in the CSF samples of late-stage and postmortem ALS patients. Decreased levels of transthyretin could suggest a failure in the transport of thyroxine and retinol/vitamin A. Consequently, this decrease would result in an inadequate sequestration of abnormally functioning proteins. Similarly, a reduction of cystatin C levels in CSF samples could indicate increased proteolysis via cysteine proteases. However, increased levels of 7B2CT could represent a reactive response to altered enzymatic activities that generate or degrade 7B2CT, or it could result from Golgi fragmentation within motor neurons during ALS [53].

Another potential ALS biomarker of tissue degeneration was studied in the blood and CSF of ALS patients. The activity of transglutaminase, a calcium-dependent enzyme that accelerates the cross-linkage of polypeptide chains by catalyzing the covalent binding of polyamines to g-glutamine residues of protein molecules, was monitored in the serum and CSF of SALS patients. There was a positive correlation between the serum enzyme activity and ALS scores. In the CSF, the enzyme activity was significantly downregulated in SALS patients after the middle disease stage with ALS clinical scores more than 250, suggesting that transglutaminase activity could be used as a general marker of neuronal degeneration during disease prognosis [54].

Exploring minimally or noninvasive samples, such as salivary samples, is also particularly interesting for the late disease stage. Chromogranin A (CgA), an endocrinological stress marker, is a soluble protein that can be measured in saliva. Salivary CgA levels have a significant positive correlation with emotional function scores on the ALSAQ-40 in middle stage ALS patients and these levels were also found upregulated in terminal ALS patients. Consequently, salivary CgA could be used as a useful quantitative biochemical marker of the affective state from symptomatic to terminal disease stages and therefore salivary CgA levels could make more effective, tailor-made psychophysiological therapies for individual ALS patients [55].

Finally, a new type of ALS biomarker is emerging from epigenetics. The connection between methylomics and transcriptomics data could reveal methylation changes in ALS tissues, such as the spinal cord or blood. Blood samples are particularly interesting because blood is a minimally invasive alternative sample source for ALS prognostic and diagnostic assessments. In particular, a recent study showed alterations in global methylation and hydroxymethylation in postmortem spinal cord samples of SALS patients. Precisey, more than a hundred genes associated with biological functions related to immune and inflammation responses were identified as differentially methylated in this study, highlighting the role of inflammatory changes in the advanced stage of the disease [56]. Epigenetics is a new field exploring potential ALS biomarkers and further studies are needed to integrate the new findings into our current knowledge. Genome-wide association studies can undoubtedly provide a better understanding of ALS.

## 4. An Update on the Therapeutic Strategies for ALS

Inflammation, apoptosis, and oxidative stress are the selected neurodegenerative pathways in ALS that are commonly tackled by therapeutic treatments.

To prevent neuronal loss and motor dysfunction and to increase survival time, potent antioxidants, such as iron chelating compounds, 2-hydroxy-5-(2,3,5,6-tetrafluoro-4-trifluoromethyl-benzylamino)-benzoic acid (Neu2000), celastrol, an antioxidant peptide (SS-31) or lipophilic metal chelators DP-109 and DP-460 and inhibitors of proapoptotic proteins, such as lithium carbonate (Li^+^), have been tested in transgenic SOD1^G93A^ mice, yielding positive results [57–61]. Similarly, in wobbler mice, another murine ALS model, treatment from 4 to 9 weeks of age with rhTNF-*α* binding protein (rhTBP-1) reduced the progression of symptoms, motor neuron loss, gliosis, and JNK/p38MAPK phosphorylation, which are two main effectors in the neuroinflammatory response [62]. Immunomodulatory agents, such as the monoclonal antibody to CD40L, a membrane glycoprotein that is primarily expressed on activated T cells, have also paved the way to the identification of cellular pathways directly involved in potential therapeutic interventions. In fact, treatment with anti-CD40L antibody reduced markers of neuroinflammation, increased the number of motor neuron cell bodies, decreased peripheral nervous system (PNS) inflammatory markers, and downregulated expression of costimulatory genes in the spinal cord of transgenic SOD1^G93A^ mice [63]. Furthermore, the use of thalidomide and its analogue lenalidomide inhibits the expression of TNF-*α* and other cytokines, showing neuroprotective properties and significantly increasing the life span of SOD1^G93A^ transgenic mice [64].

Previous studies have suggested the potential role of neurotrophic factors on motor neuron differentiation* in vitro* [65]. Combined treatment with these neuroprotective proteins can open the door to a new tool for the treatment of motor neuron diseases such as ALS. Although ciliary neurotrophic factor (CNTF), glial-derived neurotrophic factor (GDNF), insulin-like growth factor (IGF-1), and erythropoietin (EPO) improved motor behavior and reduced motor neuron loss and astrocyte/microglia activation in preclinical animal models [66, 67], clinical trials in ALS patients lacked therapeutic efficacy [68]. However, melatonin treatment has demonstrated preclinical and clinical effectiveness and high-dose melatonin could be suitable for clinical trials aimed at neuroprotection through antioxidation in ALS [69]. More recently, although the inhibition of epidermal growth factor receptor (EGFR) signaling with erlotinib might protect neurons from degeneration, no extended survival has been observed in transgenic SOD1^G93A^ mice. Consequently, the expected effect of this EGFR inhibitor* in vivo* lacked efficacy in this animal model [70]. Another novel molecular target that could provide a promising strategy for ALS is S1R, an endoplasmic reticulum- (ER-) resident receptor with chaperone-like activity. S1R can participate as a modulator in Ca^2+^ homeostasis, ER stress reaction, and apoptosis. Interestingly, mutations of the S1R gene are associated with a familial form of FTLD, which was at the same time associated with MND and a familial juvenile form of ALS. Treatment with an S1R agonist, PRE-084, improved locomotor function and motor neuron survival in presymptomatic and early-symptomatic mutant SOD1^G93A^ mice, resembling the neuroprotective effects previously tested* in vitro* [71].

Other therapeutic agents that have been mainly tested in murine ALS models are antiglutamatergic agents, such as Riluzole; the calcium regulators Verapamil and Nimodipine; creatine, a mitochondrial enhancer and antiviral therapeutic molecules such as interferon-*α* [67]. A wide range of agents associated with protein repairing pathways and chaperone activation (Arimoclomol), sodium channel blockers (Mexiletine), and others are undergoing clinical trials as shown on the main web page of the “ALS Association” (http://www.alsconsortium.org/trials.php). More in depth, Riluzol is the only drug licensed for symptomatic ALS treatment. The neuroprotective properties of Riluzol have been well documented* in vitro* in a rat ALS model, providing evidence of its neuroprotective action on motor neurons and glia after the induction of an excitotoxic stimulus [72]. Riluzol was approved for use in 1996, although 18 years later and many trials later no efficient treatment has been found to halt the neurodegenerative progression of the disease. Two randomized controlled trials showed that Riluzol reduced mortality modestly with few common side effects. Riluzol extended survival by about 11%. Since these two trials, more than 30 trials have reached negative results. Only other positive trial tested efficacy of a medication based on the combination of dextromethorphan and quinidine (AVP-923; Nuedexta). This treatment alleviated the symptoms of pseudobulbar affect in a multicenter, randomized, and controlled trial [73]. Finally, another antiglutamatergic agent that could open a new door to future therapeutic strategies in ALS patients is Gacyclidine (GK11), a noncompetitive N-methyl-D-aspartate (NMDA) receptor, which has already been used in two clinical trials for CNS lesions. Chronic treatment with a low dose of GK11 in transgenic SOD1^G93A^ mice during the early symptomatic stage improved survival and delayed locomotor function impairment [74].

## 5. Innovative Perspectives and Concluding Remarks

Gene and stem cell therapies are holding hope for an efficient ALS treatment. Regarding gene therapy, the possibility of delivering therapeutic molecules (IGF, VEGF, GDNF, BDNF, Bcl2) to damaged tissues crossing the blood-brain barrier has been made possible by the study of viral (adenovirus, adenoassociated and lentivirus) and nonviral (fragment C of tetanus toxin) vectors. These vectors are retrogradely transported to motor neurons in preclinical animal models and demonstrate promising neuroprotective effects [75–78]. Moreover, the use of small interfering RNAs (RNAi) is an alternative gene therapy approach based on the knockdown of genes that might be causing neurodegeneration, such as mutant SOD1 or Fas receptor [78, 79]. More recently and in connection with* C9ORF72* expansion, a strategy based on antisense oligonucleotides directed to* C9ORF72* transcript or repeat expansion induced amelioration in the* C9ORF72* pathogenic gain of function and in the vulnerability to excitotoxicity* in vitro* [12]. More information about relevant projects that are selected from a multidisciplinary perspective can be found on the main web page of “The Robert Packard Center for ALS Research at Johns Hopkins” (http://www.alscenter.org/als_science/research_projects/). For example, one project is currently studying the effectiveness of adenoassociated virus (AAV9) delivery to carry IGF-1 and VEGF to astrocytes.

Similar promising treatments are being carried out with stem cell therapies, which is the newest therapeutic treatment. To date, stem cell research remains at a preclinical level and the most used treatments in ALS animal models are based on neural or precursor stem cells [80] that can also be modified to release neurotrophic factors, such as GDNF [81]. Cell therapy studies based on mesenchymal stem cell (MSC) transplantation noted the potential benefit of neural induction by neurogenin 1 from transplanted MSCs in the delay of disease onset and in the enhancement of motor functions in transgenic SOD1^G93A^ mice [82]. More recently, ongoing clinical trials based on fetal-derived neural stem cell injection in ALS patients try to assess the safety and feasibility of this potential therapeutic approach into lumbar and/or cervical spinal cord regions. The preliminary results have suggested that this cell therapy is well-tolerated and pave the way for future trial phases in order to determine the therapeutic dose and efficacy [83, 84].

Although the development of therapeutic strategies that could stop or reverse ALS progression is one of our main research goals, the lack of fully validated and clinically implemented biomarkers is a challenging barrier. The heterogeneity of ALS is characterized by the involvement of many altered pathways and therefore the unknown cause of the neurodegenerative process makes the task of finding definite and specific disease biomarkers difficult. However, despite the multifactorial etiology of ALS, all of the affected proteins and genes that participate and contribute to the lethality of the disease should not be studied independently but as a whole. As in Ockham's Razor, “*do not multiply entities beyond necessity,*” when several explanations of increasing complexity are found dealing with ALS, the simplest explanation usually appears to be the most probable. At this point, the most accurate knowledge of FALS addresses SOD1 mutations and the only potential biomarkers are described for SALS. However, the new discovery of biomarkers will facilitate a better understanding of this neurodegenerative disease and will facilitate translation from animal models to patients for a definitive therapeutic approach.

## Figures and Tables

**Table 1:** Gene mutations described in FALS and SALS, their chromosome location, Gene mutations described in FALS and SALS, their chromosome location and their closely related molecular pathways.

Gene	Chromosome location	Deregulated pathway	Clinical features
**ANG** angiogenin	14q11.2	Angiogenesis pathway	Sporadic ALS

**APEX1** DNA repair enzyme endonuclease	14q11.2-q12	Oxidative stress	Sporadic ALS

**C9orf72** Chromosome 9 open reading frame 72	9p21.2	RNA metabolism	Familial ALS found in sporadic ALS, FTD

**CHMP2B** chromatin modifying protein 2B	3q11.2	Endosomal trafficking	Familial ALS, FTD

**CNTF** ciliary neurotrophic factor	11q12.2	Neurotrophic factor, inflammation	Sporadic ALS

**DCTN1** dynactin	2q13	Deregulation of retrograde axonal transport of vesicles and organelles	Lower motor neuron disorder

**FIG4** SAC domain-containing protein	6q21	Trafficking endosomal vesicles	Slow progression juvenile ALS

**FUS/TLS** DNA/RNA-binding protein	16q12.1-12.2	Transcriptional regulation, RNA splicing and transport	Familial and sporadic ALS

**HFE** haemochromatosis	6q21.3	Disruption of iron metabolism	Sporadic ALS

**MAPT** microtubule-associated protein tau	17q21	Neurofilament structure and axonal integrity alterations	ALS disorder with Parkinsonism and dementia

**NEFL, NEFM, NEFH** neurofilaments chains	8q21; 22q12.1-q13.1	Neurofilament structure and axonal integrity alterations	Sporadic ALS

**PGRN** progranulin	17q21.32	Induction of ubiquitin-positive processes, TAU-negative FTD	Sporadic ALS

**PON** paraoxonase	7q21.2-q22.1	Failure in the detoxification of organophosphate and neurotoxins	Sporadic ALS

**PRPH** peripherin	12q12-q13	Filament alterations in autonomic nerves and peripheral sensory neurons	Sporadic ALS

**SETX** senataxin	9q34	DNA and RNA processing	Slow progression juvenile ALS

**SMN** survival motor neuron gene	5q13.3	Child-onset spinal muscular atrophy linked to SMN1 mutations	Lower motor neuron disorder

**SOD-1** copper/zinc superoxide-dismutase-1	21q22.1	Upregulation of protein tyrosine-nitration Improper metal ion binding Upregulation of proinflammatory cytokines Formation of intracellular aggregates Mitochondrial dysfunction Reduced expression of glutamate transporters Deregulation of calcium homeostasis Downregulation of intracortical inhibitory processes Cell death activation Deregulation of Na+ and K+ cell gradients Slowing of anterograde transport	Familial ALS

**SPG11** spatacsin	15q15.1-21.1	Axonal transport	Slow progression juvenile ALS

**TARDPB** tar DNA-binding protein	1q36.22	Neurodegeneration of neurons, oligodendroglia, and astrocytes	Familial and sporadic ALS

**VAPB** vesicle-associated membrane protein-associated protein B	20q13.33	Induction of the unfolded protein response altered transport and secretion pathways	Familial ALS

**VEGF** vascular endothelial cell growth factor	6p12	Disease severity linked to SMN2 mutations angiogenesis, permeability blood vessels	Sporadic ALS

## References

[1] Brooks BR, Miller RG, Swash M, Munsat TL (2000). El Escorial revisited: revised criteria for the diagnosis of amyotrophic lateral sclerosis. *Amyotrophic Lateral Sclerosis*.

[2] Lomen-Hoerth C, Murphy J, Langmore S, Kramer JH, Olney RK, Miller B (2003). Are amyotrophic lateral sclerosis patients cognitively normal?. *Neurology*.

[3] Rusina R, Ridzon P, Kulist'ák P (2010). Relationship between ALS and the degree of cognitive impairment, markers of neurodegeneration and predictors for poor outcome. A prospective study. *European Journal of Neurology*.

[4] Ringholz GM, Appel SH, Bradshaw M, Cooke NA, Mosnik DM, Schulz PE (2005). Prevalence and patterns of cognitive impairment in sporadic ALS. *Neurology*.

[5] Hays AP, Roxas A, Sadia SA (1990). A monoclonal IgA in patient with amyotrophic lateral sclerosis reacts with neurofilaments and surface antigen on neuroblastoma cells. *Journal of Neuropathology and Experimental Neurology*.

[6] Wijesekera LC, Leigh PN (2009). Amyotrophic lateral sclerosis. *Orphanet Journal of Rare Diseases*.

[7] Katsuno M, Tanaka F, Sobue G (2012). Perspectives on molecular targeted therapies and clinical trials for neurodegenerative diseases. *Journal of Neurology, Neurosurgery and Psychiatry*.

[8] Jones AR, Woollacott I, Shatunov A (2013). Residual association at C9orf72 suggests an alternative amyotrophic lateral sclerosis-causing hexanucleotide repeat. *Neurobiology of Aging*.

[9] Chiò A, Battistini S, Calvo A (2014). Genetic counselling in ALS: facts, uncertainties and clinical suggestions. *Journal of Neurology, Neurosurgery and Psychiatry*.

[10] Rosen DR, Siddique T, Patterson D (1993). Mutations in Cu/Zn superoxide dismutase gene are associated with familial amyotrophic lateral sclerosis. *Nature*.

[11] Pasinelli P, Brown RH (2006). Molecular biology of amyotrophic lateral sclerosis: Insights from genetics. *Nature Reviews Neuroscience*.

[12] Su XW, Broach JR, Connor JR (2014). Genetic heterogeneity of ALS: implications for clinical practice and research. *Muscle & Nerve*.

[13] Cluskey S, Ramsden DB (2001). Mechanisms of neurodegeneration in amyotrophic lateral sclerosis. *Molecular Pathology*.

[14] Schymick JC, Talbot K, Traynor BJ (2007). Genetics of sporadic amyotrophic lateral sclerosis. *Human Molecular Genetics*.

[15] Graber DJ, Hickey WF, Harris BT (2010). Progressive changes in microglia and macrophages in spinal cord and peripheral nerve in the transgenic rat model of amyotrophic lateral sclerosis. *Journal of Neuroinflammation*.

[16] Vucic S, Kiernan MC (2009). Pathophysiology of neurodegeneration in familial amyotrophic lateral sclerosis. *Current Molecular Medicine*.

[17] Chancellor AM, Slattery JM, Fraser H, Swingler RJ, Holloway SM, Warlow CP (1993). The prognosis of adult-onset motor neuron disease: a prospective study based on the Scottish motor neuron disease register. *Journal of Neurology*.

[18] Burgunder J-M, Schöls L, Baets J (2011). EFNS guidelines for the molecular diagnosis of neurogenetic disorders: motoneuron, peripheral nerve and muscle disorders. *European Journal of Neurology*.

[19] Johnston CA, Stanton BR, Turner MR (2006). Amyotrophic lateral sclerosis in an urban setting: a population based study of inner city London. *Journal of Neurology*.

[20] Bowser R, Lacomis D (2009). Applying proteomics to the diagnosis and treatment of ALS and related diseases. *Muscle and Nerve*.

[21] Lomen-Hoerth C (2008). Amyotrophic lateral sclerosis from bench to bedside. *Seminars in Neurology*.

[22] Khader SM, Greiner FG (1999). Neuroradiology case of the day. *Radiographics*.

[23] Bondy SC, Lee DK (1993). Oxidative stress induced by glutamate receptor agonists. *Brain Research*.

[24] Menzies FM, Cookson MR, Taylor RW (2002). Mitochondrial dysfunction in a cell culture model of familial amyotrophic lateral sclerosis. *Brain*.

[25] Vucic S, Howells J, Trevillion L, Kiernan MC (2006). Assessment of cortical excitability using threshold tracking techniques. *Muscle and Nerve*.

[26] Vucic S, Nicholson GA, Kiernan MC (2008). Cortical hyperexcitability may precede the onset of familial amyotrophic lateral sclerosis. *Brain*.

[27] de Carvalho M, Dengler R, Eisen A (2008). Electrodiagnostic criteria for diagnosis of ALS. *Clinical Neurophysiology*.

[28] Makki AA, Benatar M (2007). The electromyographic diagnosis of amyotrophic lateral sclerosis: does the evidence support the El Escorial criteria?. *Muscle and Nerve*.

[29] Costa J, Swash S, de Carvalho M (2012). Awaji criteria for the diagnosis of Amyotrophic Lateral Sclerosis: a systematic review. *Archives of Neurology*.

[30] Pinto AC, Alves M, Nogueira A (1999). Can amyotrophic lateral sclerosis patients with respiratory insufficiency exercise?. *Journal of the Neurological Sciences*.

[31] Chiò A, Calvo A, Ilardi A (2009). Lower serum lipid levels are related to respiratory impairment in patients with ALS. *Neurology*.

[32] Azuaje F (2010). *Bioinformatics and Biomarker Discovery: “Omic” Data Analysis for Personalized Medicine*.

[33] Balendra R, Jones A, Jivraj N (2014). Estimating clinical stage of amyotrophic lateral sclerosis from the ALS Functional Rating Scale. *Amyotrophic Lateral Sclerosis and Frontotemporal Degeneration*.

[34] Chiò A, Hammond ER, Mora G (2013). Development and evaluation of a clinical staging system for amyotrophic lateral sclerosis. *Journal of Neurology, Neurosurgery & Psychiatry*.

[35] El Mendili M-M, Cohen-Adad J, Pelegrini-Issac M (2014). Multi-parametric spinal cord MRI as potential progression marker in amyotrophic lateral sclerosis. *PLoS ONE*.

[36] Dupuis L, Gonzalez de Aguilar J-L, di Scala F (2002). Nogo provides a molecular marker for diagnosis of amyotrophic lateral sclerosis. *Neurobiology of Disease*.

[37] Fergani A, Dupuis L, Jokic N (2005). Reticulons as markers of neurological diseases: focus on amyotrophic lateral sclerosis. *Neurodegenerative Diseases*.

[38] Karnezis T, Mandemakers W, McQualter JL (2004). Reticulons as markers of neurological diseases: focus on amyotrophic lateral sclerosis. *Nature Neuroscience*.

[39] Jokic N, Gonzalez De Aguilar J, Pradat P (2005). Nogo expression in muscle correlates with amyotrophic lateral sclerosis severity. *Annals of Neurology*.

[40] Zhao Z, Lange DJ, Ho L (2008). Vgf is a novel biomarker associated with muscle weakness in amyotrophic lateral sclerosis (ALS), with a potential role in disease pathogenesis. *International Journal of Medical Sciences*.

[41] Keller AF, Gravel M, Kriz J (2009). Live imaging of amyotrophic lateral sclerosis pathogenesis: disease onset is characterized by marked induction of GFAP in schwann cells. *GLIA*.

[42] Zhang X, Chen S, Li L, Wang Q, Le W (2010). Decreased level of 5-methyltetrahydrofolate: a potential biomarker for pre-symptomatic amyotrophic lateral sclerosis. *Journal of the Neurological Sciences*.

[43] Philips T, de Muynck L, Thu HNT (2010). Microglial upregulation of progranulin as a marker of motor neuron degeneration. *Journal of Neuropathology and Experimental Neurology*.

[44] Gerber YN, Sabourin J, Rabano M, Vivanco MDM, Perrin FE (2012). Early functional deficit and microglial disturbances in a mouse model of amyotrophic lateral sclerosis. *PLoS ONE*.

[45] Almer G, Vukosavic S, Romero N, Przedborski S (1999). Inducible nitric oxide synthase up-regulation in a transgenic mouse model of familial amyotrophic lateral sclerosis. *Journal of Neurochemistry*.

[46] Evans MC, Serres S, Khrapitchev AA (2014). T2-weighted MRI detects presymptomatic pathology in the SOD1 mouse model of ALS. *Journal of Cerebral Blood Flow & Metabolism*.

[47] Nicaise C, Mitrecic D, Demetter P (2009). Impaired blood-brain and blood-spinal cord barriers in mutant SOD1-linked ALS rat. *Brain Research*.

[48] Soon CPW, Crouch PJ, Turner BJ (2010). Serum matrix metalloproteinase-9 activity is dysregulated with disease progression in the mutant SOD1 transgenic mice. *Neuromuscular Disorders*.

[49] Iłzecka J, Stelmasiak Z, Dobosz B (2001). Matrix metalloproteinase-9 (MMP-9) activity in cerebrospinal fluid of amyotrophic lateral sclerosis patients. *Neurologia i Neurochirurgia Polska*.

[50] Shinozawa T, Urade Y, Maruyama T, Watabe D (2011). Tetranor PGDM analyses for the amyotrophic lateral sclerosis: positive and simple diagnosis and evaluation of drug effect. *Biochemical and Biophysical Research Communications*.

[51] Boylan K, Yang C, Crook J (2009). Immunoreactivity of the phosphorylated axonal neurofilament H subunit (pNF-H) in blood of ALS model rodents and ALS patients: evaluation of blood pNF-H as a potential ALS biomarker. *Journal of Neurochemistry*.

[52] Calvo AC, Manzano R, Atencia-Cibreiro G (2012). Genetic biomarkers for ALS disease in transgenic SOD1 G93A mice. *PLoS ONE*.

[53] Ranganathan S, Williams E, Ganchev P (2005). Proteomic profiling of cerebrospinal fluid identifies biomarkers for amyotrophic lateral sclerosis. *Journal of Neurochemistry*.

[54] Fujita K, Honda M, Hayashi R (1998). Transglutaminase activity in serum and cerebrospinal fluid in sporadic amyotrophic lateral sclerosis: a possible use as an indicator of extent of the motor neuron loss. *Journal of the Neurological Sciences*.

[55] Obayashi K, Sato K, Shimazaki R (2008). Salivary chromogranin A: useful and quantitative biochemical marker of affective state in patients with amyotrophic lateral sclerosis. *Internal Medicine*.

[56] Figueroa-Romero C, Hur J, Bender DE (2012). Identification of epigenetically altered genes in sporadic amyotrophic lateral sclerosis. *PLoS ONE*.

[57] Petri S, Kiaei M, Damiano M (2006). Cell-permeable peptide antioxidants as a novel therapeutic approach in a mouse model of amyotrophic lateral sclerosis. *Journal of Neurochemistry*.

[58] Jin HS, Sung IC, Hyang RL (2007). Concurrent administration of Neu2000 and lithium produces marked improvement of motor neuron survival, motor function, and mortality in a mouse model of amyotrophic lateral sclerosis. *Molecular Pharmacology*.

[59] Kiaei M, Kipiani K, Petri S, Chen J, Calingasan NY, Beal MF (2006). Celastrol blocks neuronal cell death and extends life in transgenic mouse model of amyotrophic lateral sclerosis. *Neurodegenerative Diseases*.

[60] Petri S, Calingasan NY, Alsaied OA (2007). The lipophilic metal chelators DP-109 and DP-460 are neuroprotective in a transgenic mouse model of amyotrophic lateral sclerosis. *Journal of Neurochemistry*.

[61] Benkler C, Offen D, Melamed E (2010). Recent advances in amyotrophic lateral sclerosis research: perspectives for personalized clinical application. *The EPMA Journal*.

[62] Bigini P, Repici M, Cantarella G (2008). Recombinant human TNF-binding protein-1 (rhTBP-1) treatment delays both symptoms progression and motor neuron loss in the wobbler mouse. *Neurobiology of Disease*.

[63] Lincecum JM, Vieira FG, Wang MZ (2010). From transcriptome analysis to therapeutic anti-CD40L treatment in the SOD1 model of amyotrophic lateral sclerosis. *Nature Genetics*.

[64] Kiaei M, Petri S, Kipiani K (2006). Thalidomide and lenalidomide extend survival in a transgenic mouse model of amyotrophic lateral sclerosis. *Journal of Neuroscience*.

[65] Zurn AD, Winkel L, Menoud A (1996). Combined effects of GDNF, BDNF, and CNTF on motoneuron differentiation in vitro. *Journal of Neuroscience Research*.

[66] Mennini T, de Paola M, Bigini P (2006). Nonhematopoietic erythropoietin derivatives prevent motoneuron degeneration in vitro and in vivo. *Molecular Medicine*.

[67] Goodall EF, Morrison KE (2006). Amyotrophic lateral sclerosis (motor neuron disease): proposed mechanisms and pathways to treatment. *Expert Reviews in Molecular Medicine*.

[68] Kalra S, Genge A, Arnold DL (2003). A prospective, randomized, placebo-controlled evaluation of corticoneuronal response to intrathecal BDNF therapy in ALS using magnetic resonance spectroscopy: feasibility and results. *Amyotrophic Lateral Sclerosis and Other Motor Neuron Disorders*.

[69] Weishaupt JH, Bartels C, Pölking E (2006). Reduced oxidative damage in ALS by high-dose enteral melatonin treatment. *Journal of Pineal Research*.

[70] Le Pichon CE, Dominguez SL, Solanoy H (2013). EGFR inhibitor erlotinib delays disease progression but does not extend survival in the SOD1 mouse model of ALS. *PLoS ONE*.

[71] Peviani M, Salvaneschi E, Bontempi L (2014). Neuroprotective effects of the Sigma-1 receptor (S1R) agonist PRE-084, in a mouse model of motor neuron disease not linked to SOD1 mutation. *Neurobiology of Disease*.

[72] Cifra A, Nani F, Nistri A (2011). Riluzole is a potent drug to protect neonatal rat hypoglossal motoneurons in vitro from excitotoxicity due to glutamate uptake block. *European Journal of Neuroscience*.

[73] Gordon PH (2013). Amyotrophic lateral sclerosis: an update for 2013 clinical features, pathophysiology, management and therapeutic trials. *Aging and Disease*.

[74] Gerber YN, Privat A, Perrin FE (2013). Gacyclidine improves the survival and reduces motor deficits in a mouse model of amyotrophic lateral sclerosis. *Frontiers in Cellular Neuroscience*.

[75] Azzouz M, Ralph GS, Storkebaum E (2004). VEGF delivery with retrogradely transported lentivector prolongs survival in a mouse ALS model. *Nature*.

[76] Kaspar BK, Lladó J, Sherkat N, Rothstein JD, Gage FH (2003). Retrograde viral delivery of IGF-1 prolongs survival in a mouse ALS model. *Science*.

[77] Ciriza J, Moreno-Igoa M, Calvo AC (2008). A genetic fusion GDNF-C fragment of tetanus toxin prolongs survival in a symptomatic mouse ALS model. *Restorative Neurology and Neuroscience*.

[78] Moreno-Igoa M, Calvo AC, Penas C (2010). Fragment C of tetanus toxin, more than a carrier. Novel perspectives in non-viral ALS gene therapy. *Journal of Molecular Medicine*.

[79] Locatelli F, Corti S, Papadimitriou D (2007). Fas small interfering RNA reduces motoneuron death in amyotrophic lateral sclerosis mice. *Annals of Neurology*.

[80] Corti S, Locatelli F, Papadimitriou D (2007). Neural stem cells LewisX + CXCR4 + modify disease progression in an amyotrophic lateral sclerosis model. *Brain*.

[81] Suzuki M, McHugh J, Tork C (2007). GDNF secreting human neural progenitor cells protect dying motor neurons, but not their projection muscule, in a rat model of familial ALS. *PLoS ONE*.

[82] Choi C, Lee Y, Kim H, Kim SH, Suh-Kim H (2013). Neural induction with neurogenin 1 enhances the therapeutic potential of mesenchymal stem cells in an amyotrophic lateral sclerosis mouse model. *Cell Transplantation*.

[83] Glass JD, Boulis NM, Johe K (2012). Lumbar intraspinal injection of neural stem cells in patients with amyotrophic lateral sclerosis: results of a phase I trial in 12 patients. *Stem Cells*.

[84] Feldman EL, Boulis NM, Hur J (2014). Intraspinal neural stem cell transplantation in amyotrophic lateral sclerosis: phase 1 trial outcomes. *Annals of Neurology*.

